# Supplementary material for: Sustained-input switches for transcription factors and microRNAs are central building blocks of eukaryotic gene circuits
Source: Genome Biol. 2013 Aug 23;14(8):R85. doi: 10.1186/gb-2013-14-8-r85 (PMC4054853; doi:10.1186/gb-2013-14-8-r85)
Supplement: Additional file 5 — HTML Browsable Motif Output. Zipped folder containing all WaRSwap and FANMOD motif output, viewable in a web browser. [file gb-2013-14-8-r85-S5.ZIP › HTML_browsable_motif_output/WaRSwap_all_tair9/sigs_WSWRSLDraw-2000_all.pvals.heatmaps.html/motif_id_12_000001101_tftype_all_upstream_-1000_0.html]

```
BG_MODEL = Rnew2
MOTIF_ID = 12_000001101
TF_TYPE = all
UPSTREAM = -1000_0


PVals
FN_0.2	FN_0.4	FN_0.6	FN_0.8
dg_60.genes	0.015	0.443	0.29	0.562
dg_70.genes	0.025	0.539	0.327	0.617
dg_80.genes	0.003	0.141	0.08	0.69

ZScores
FN_0.2	FN_0.4	FN_0.6	FN_0.8
dg_60.genes	2.645	0.084	0.526	-0.277
dg_70.genes	2.303	-0.186	0.396	-0.399
dg_80.genes	3.776	1.089	1.476	-0.589

StDevs
FN_0.2	FN_0.4	FN_0.6	FN_0.8
dg_60.genes	105.651	96.485	64.074	36.284
dg_70.genes	107.322	92.551	62.619	36.016
dg_80.genes	86.119	73.514	47.491	25.614
```
